# *Sm*MYC2a and *Sm*MYC2b played similar but irreplaceable roles in regulating the biosynthesis of tanshinones and phenolic acids in *Salvia miltiorrhiza*

**DOI:** 10.1038/srep22852

**Published:** 2016-03-07

**Authors:** Yangyun Zhou, Wei Sun, Junfeng Chen, Hexin Tan, Ying Xiao, Qing Li, Qian Ji, Shouhong Gao, Li Chen, Shilin Chen, Lei Zhang, Wansheng Chen

**Affiliations:** 1 Department of Pharmacy, Changzheng Hospital, Second Military Medical University, Shanghai 200003, China; 2 Institute of Chinese Materia Medica, China Academy of Chinese Medicinal Sciences, Beijing 100700, China; 3 Department of Pharmaceutical Botany, School of Pharmacy, Second Military Medical University, Shanghai 200433, China

## Abstract

*Salvia miltiorrhiza* Bunge, which contains tanshinones and phenolic acids as major classes of bioactive components, is one of the most widely used herbs in traditional Chinese medicine. Production of tanshinones and phenolic acids is enhanced by methyl jasmonate (MeJA). Transcription factor MYC2 is the switch of jasmontes signaling in plants. Here, we focused on two novel JA-inducible genes in *S. miltiorrhiza*, designated as *SmMYC2a* and *SmMYC2b*, which were localized in the nucleus. *Sm*MYC2a and *Sm*MYC2b were also discovered to interact with *Sm*JAZ1 and *Sm*JAZ2, implying that the two MYC2s might function as direct targets of JAZ proteins. Ectopic RNA interference (RNAi)-mediated knockdown experiments suggested that *Sm*MYC2a/b affected multiple genes in tanshinone and phenolic acid biosynthetic pathway. Besides, the accumulation of tanshinones and phenolic acids was impaired by the loss of function in *SmMYC2a/b*. Meanwhile, *Sm*MYC2a could bind with an E-box motif within *SmHCT6* and *SmCYP98A14* promoters, while *Sm*MYC2b bound with an E-box motif within *SmCYP98A14* promoter, through which the regulation of phenolic acid biosynthetic pathway might achieve. Together, these results suggest that *Sm*MYC2a and *Sm*MYC2b are JAZ-interacting transcription factors that positively regulate the biosynthesis of tanshinones and Sal B with similar but irreplaceable effects.

*Salvia miltiorrhiza* Bunge, named Danshen in China, is a widely used traditional Chinese medicine with a remarkable clinical effect on cardiovascular diseases, cerebrovascular, hyperlipidemia and acute ischemic stroke^1^,^2^,^3^. Pharmacological investigations indicate that the active ingredients of *S. miltiorrhiza* are lipophilic tanshinones and hydrophilic phenolic acids^3^,^4^,^5^. Jasmonate acid (JA) and its oxylipin derivatives, collectively called jasmonates, are phytohormones that play an integral role in various defense responses, leading to the accumulation of secondary metabolites^6^. The biosynthesis of JA has been extensively studied and well-defined^7^. Under bioactive jasmonate stress, JA is conjugated to isoleucine. Jasmonoyl-L-isoleucine (JA-Ile) is formed and perceived by Coronatine Insensitive 1 (COI1) protein to form Skp1/Cullin1/F-box protein COI1 (SCF^COI1^) complexes. Afterwards, Jasmonate ZIM-domain (JAZ) proteins are recruited, ubiquitinated and subsequently degraded, leading to the release and activation of MYC2 proteins^8^,^9^,^10^. However, under stress-free growth conditions, Jasmonate ZIM-domain (JAZ) proteins recruit its co-repressors, such as TOPLESS (TPL) or TPL-related proteins (TRPs)^11^,^12^,^13^, leading to the repression of JA responses.

Methyl jasmonate (MeJA) has been commonly used as an elicitor to explore the regulatory mechanisms underlying the biosynthesis of active compounds^14^. There have been reports indicating that the accumulation of tanshinone IIA and Salvianolic acid B (Sal B) in *S. miltiorrhiza* hairy root cultures is accelerated by applying exogenous MeJA or improving endogenous JA^15^,^16^,^17^. Multiple MeJA-responsive transcription factors (TFs) and genes involved in the regulation and biosynthesis of tanshinones and phenolic acids pathways were previously mined without advanced functional verifications yet^18^. The specific role that MeJA plays in the activation of TFs which regulate a wide array of pathway genes is still veiled in *S. miltiorrhiza*. Whether MYC2 regulates the biosynthesis of tanshinones and Sal B still remains poorly understood.

Here, we focused on two MYC2 genes, *SmMYC2a* and *SmMYC2b*, which belonged to bHLH gene family. *SmMYC2a* and *SmMYC2b* turned out to be positive JA-responsive genes, which not only affected multiple genes in tanshinones and Sal B biosynthetic pathway, but also dramatically influenced the output of tanshinones and Sal B. In electrophoretic mobility shift assay (EMS assay), *Sm*MYC2a could bind with an E-box motif within promoters of hydroxy-cinnamoyl transferase 6 (*SmHCT6*) and cytochrome P450-dependent monooxygenase 98A14 (*SmCYP98A14*), while *Sm*MYC2b bound with an E-box motif within *SmCYP98A14* promoter. These results indicated that *Sm*MYC2a and *Sm*MYC2b were positive regulators that strongly regulated pathway genes in the biosynthesis of tanshinones and phenolic acids.

## Results

### Molecular Cloning, phylogenetic analysis and organ expression pattern of *SmMYC2a* and *SmMYC2b*

**Figure 1:**
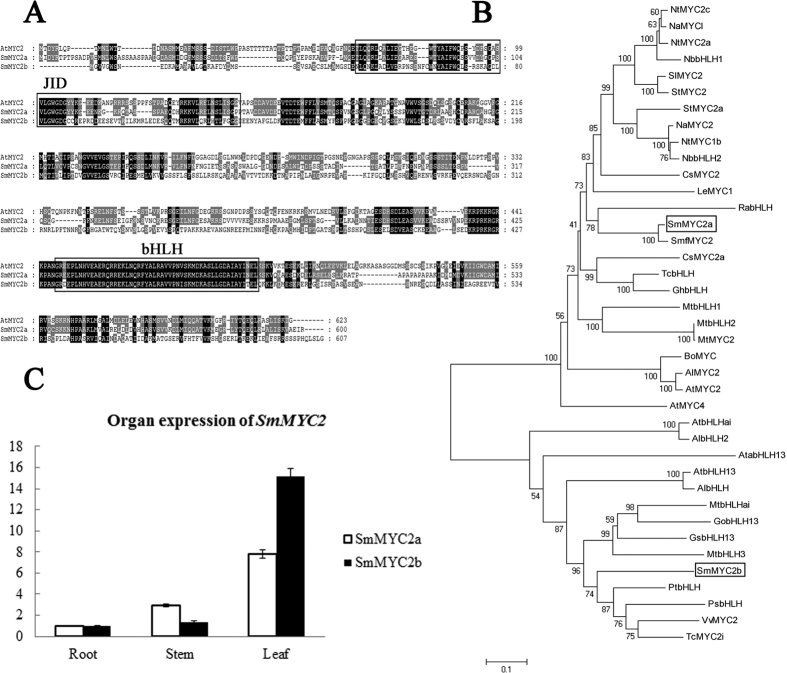
Multiple alignment, phylogenetic analysis and organ expression analysis of *SmMYC2a* and *SmMYC2b*. (**A**) Alignment of *Sm*MYC2a, *Sm*MYC2b and *Arabidopsis* MYC2 homologs. *At*MYC2 (GenBank Accession: NM_102998.3) amino acid sequence was displayed. bHLH domain was boxed. Black highlighted residues indicated identical residues and gray boxes indicated similar residues. (**B**) Phylogenetic tree was constructed on MEGA using neighbor-joining method. Bootstrap values were obtained for 1000 replications. NJ tree included *Sm*MYC2s and 37 bHLH proteins from other plant species (Supplementary Table S1). The bars represent evolutionary distance. (**C**) Organ expression pattern of *Sm*MYC2a and *Sm*MYC2b. Transcript abundance of each MYC2 is normalized to actin as control using 2^−△△Ct^ method.

Based on the built MeJA-induced gene expression profiling database of *S. miltiorrhiza*^18^, two JA-responsive MYC2 members, *SmMYC2a* and *SmMYC2b* were successfully amplified. The genomic sequence of *SmMYC2a* contained an 1803 bp ORF without introns, and *SmMYC2b* contained an ORF of 1809 bp without introns. Meanwhile, the two MYC2s contained conserved bHLH domain and JAZ interaction domain (JID) (Fig. 1A), which defined bHLH family. Alignment of *Sm*MYC2a, *Sm*MYC2b and *Arabidopsis* MYC2 was displayed at amino acid level, indicating that *Sm*MYC2a and *Sm*MYC2b shared a 53% and 47% identity with *At*MYC2 respectively. For evolutionary relationship analysis, two *Sm*MYC2s, together with 37 bHLH proteins from various plant species, were used to construct a Neighbour-joining (NJ) tree at the amino acid level. The dendrogram (Fig. 1B) showed that the selected MYC2s were clustered into two groups, group I and group II. *Sm*MYC2a was clustered into a subgroup, which belonged to group I, with *Smf*MYC2, while *Sm*MYC2b belonged to group II. Organ expression analysis was carried out to study the expression pattern of *SmMYC2s*. Both *SmMYC2a* and *SmMYC2b* were discovered to be constitutively expressed in roots, stems and leaves. Meanwhile, both genes were significantly highly expressed in leaves, followed by stems and roots (Fig. 1C).

### *Sm*MYC2a and *Sm*MYC2b were localized in the nucleus

**Figure 2:**
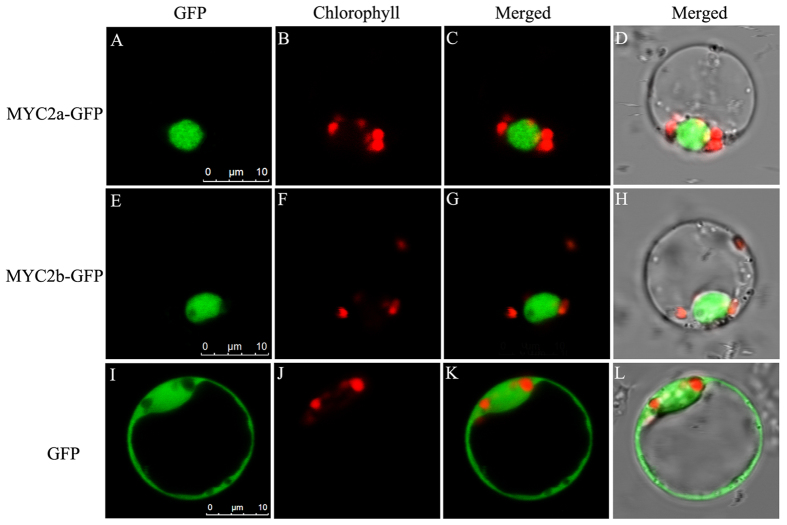
*SmMYC2a* and *SmMYC2b* were localized in nucleus. (**A**) A rice protoplast expressing MYC2a-GFP showed green fluorescent signals in the nucleus. (**B**) The same protoplast cell of (A) showing the chlorophyll autofluorescence signal in the plastids. (**C**) The merged signal of (**A**,**B**). (**D**) The same protoplast of (**C**) under white light. Bars = 10 μm. (**E**) A rice protoplast which expressed MYC2b-GFP showed the green fluorescent signal in the nucleus. (**F**) The same rice protoplast of (**E**) expressing red fluorescent signal in the plastids. (**G**) The merged signal of (**E**,**F**). (**H**) The same protoplast of (**G**) under white light. Bars = 10 μm. (**I**) Rice protoplast that expressed free GFP showed green fluorescent signals as control. (**J**) The same rice protoplast cell of (**I**) showing red fluorescent signals. (**K**) The merged signal of (**I,K**). (**L**) The same protoplast of (**K**) under white light. Bars = 10 μm.

To investigate the subcellular localization of *Sm*MYC2a *and Sm*MYC2b, the constructed *pCAMBIA1301-GFP-SmMYC2a/b* vector was transformed into prepared rice protoplasts. As shown in Fig. 2, the free green fluorescent protein (GFP) was detected in the nucleoplasm and cytoplasm. By contrast, the two TFs presented exclusively nuclear fluorescent signals in the observed protoplasts, indicating the localization in the nucleus. The nucleus localization characteristic confirmed the expectation of *Sm*MYC2a and *Sm*MYC2b as TFs.

### *Sm*MYC2a and *Sm*MYC2b formed complexes with *Sm*JAZ1 and *Sm*JAZ2 repressors in yeast two-hybrid assay

**Figure 3:**
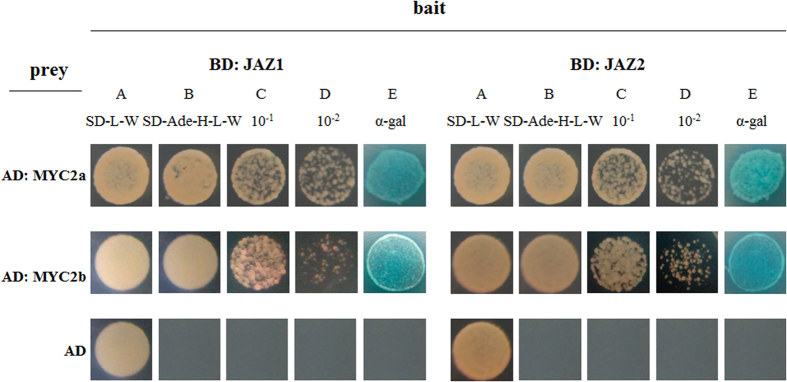
The interactions of *Sm*MYC2s and *Sm*JAZs *in vitro*. (**A**) AH109 yeast cells were grown on SD medium lacking leucine and tryptophan. (**B**) SD medium lacking adenine, histidine, leucine and tryptophan. (**C**) The 10-fold dilutions of yeast cells of (**B**). (**D**) 100-fold dilutions of yeast cells of (**B**). (**E**) Assayed for MEL1 activity.

Yeast two-hybrid (Y2H) assay using JAZ1 and JAZ2 as baits was implemented to investigate the interactive ability of *Sm*MYC2 with *Sm*JAZ repressors. The control strain, containing pAD-GAL4 (AD) and pBD-GAL4 (BD), could grow on double selection medium (SD/–Leu/–Trp) but not capable of growing on quadruple selection medium (SD/–Ade/–His/–Leu/–Trp) (Fig. 3), indicating the feasibility of the system. Strains where *pGADT7-MYC2a/b* and *pGBKT7* vectors were transformed showed same growing states with above control strains, suggesting that *Sm*MYC2a and *Sm*MYC2b were not capable of auto-activating the reporting gene (Supplementary Fig. S1). However, transformed yeast cells which harbored pAD-GAL4-MYC2a/b (AD-MYC2a/b) plus pBD-GAL4-*Sm*JAZ1/2 (BD-JAZ1/2) grew well on both double and quadruple selection medium, suggesting *in vitro* interactions between MYC2 and JAZs. Subsequently, α-galactosidase (α-gal) assay was applied to reconfirm their interactions. The appearance of blue colonies was observed, which was due to the activation of MEL1 reporter, indicating the binding of MYC2-JAZ. The Y2H result demonstrated that *Sm*MYC2 regulators could interact with *Sm*JAZ repressors.

### *Sm*MYC2a and *Sm*MYC2b were inducible by MeJA in *S. miltiorrhiza* hairy roots

**Figure 4:**
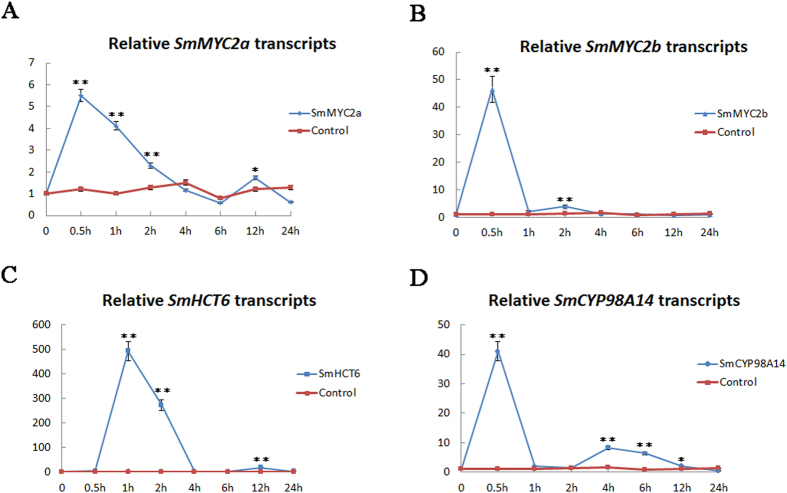
MeJA-induced transcript level changes of *SmMYC2a* and *SmMYC2b*. Each data point was the average of three biological replicates. Bars indicated SD. Asterisks indicated statistically significant differences when compared with 0 h. “*” indicated *p* < 0.05 and “**” indicated *p* < 0.01. (**A**,**B**) showed qRT-PCR analysis of *SmMYC2a/b* expression at various times after MeJA treatment in *S. miltiorrhiza* hairy roots. (**C**,**D**) respectively showed qRT-PCR analysis of *SmCYP98A14* and *SmHCT6* expression at various times following MeJA treatment.

Both *SmMYC2a* and *SmMYC2b* were induced with exogenous MeJA, reaching a maximal level at 0.5 h after treatment. The transcript levels of *SmMYC2a* and *SmMYC2b* increased rapidly within 30 min (Fig. 4). Afterwards, the variation trends for transcript levels of *SmMYC2a* and *SmMYC2b* slightly differed. Transcript level of *SmMYC2a* declined slowly till 4 h and a minor increase occurred at 12 h post-treatment with MeJA. However, transcript level of *SmMYC2b* decreased sharply till 1 h and the second peak appeared at 2 h after MeJA treatment. The variation profiling of *SmHCT6* and *SmCYP98A14*, two pathway genes of phenolic acid biosynthesis, was characterized in Fig. 4. The expression level of *SmHCT6* and *SmCYP98A14* increased 492.4 and 40.2 folds respectively at 0.5 h. Generally speaking, the variation trends were in parallel with changes in *SmMYC2a* and *SmMYC2b* transcript levels.

### Expression of critical pathway genes was affected in *MYC2-RNAi* hairy roots

**Figure 5:**
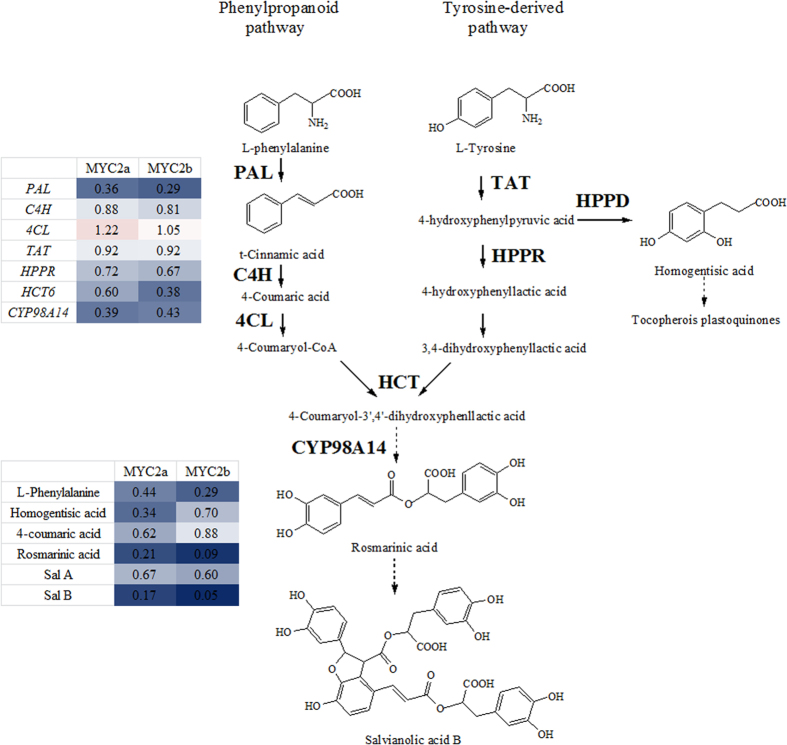
The production of phenolic acids and the expression of biosynthetic genes were repressed in *MYC2-RNAi* hairy roots compared with control. MYC2a, *SmMYC2a* suppressed hairy roots. MYC2b, *SmMYC2b* suppressed hairy roots. PAL, phenylalanine ammonialyase; C4H, cinnamic acid -4-hydroxylase; 4CL, hydroxycinnamate, coenzyme A ligase; TAT, tyrosine aminotransferase; HPPR, 4-hydroxyphenylpyruvate reductase; HPPD, 4-hydroxy phenyl pyruvate dioxygenase; HCT, hydroxy-cinnamoyl transferase; CYP98A14, cytochrome P450 monooxygenase 98A14. Red indicated enhanced expression or production; blue indicated repressed expression or production; white indicated the expression of control; darker color indicated more significant change. The relative expression values were converted into color array by Microsoft Excel software (version 2010). All the values were ratios of average gene expression level or compounds content in *MYC2-RNAi* hairy roots to control.

**Figure 6:**
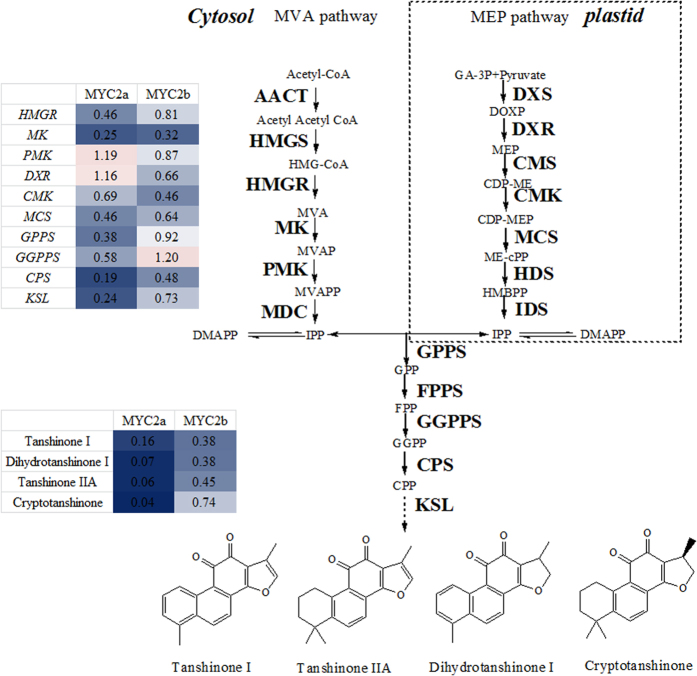
The production of tanshinones and the expression of biosynthetic genes were repressed in transgenic *SmMYC2a/b* suppressed hairy roots compared with control. MYC2a, *SmMYC2a* suppressed hairy roots. MYC2b, *SmMYC2b* suppressed hairy roots. AACT, acetyl-CoA C-acetyltransferase; CDP-ME, 4-(cytidine 5’-diphospho)-2-C-methyl- D-erythritol; CDP-ME2P, 2-phospho-4-(cytidine 5’-diphospho)-2-C-methyl-D-erythritol; CMK, 4-(cytidine 5’-diphospho)-2-C-methyl- D-erythritol kinase; CPS, copalyl diphosphate synthase; DMAPP, dimethylallyl diphosphate; DXP, 1-deoxy-D-xylulose 5-phosphate; DXR, 1-deoxy-D-xylulose 5-phosphate reductoisomerase; DXS, 1-deoxy-D-xylulose 5-phosphate synthase; FPP, farnesyldiphosphate; FPPS, farnesyl diphosphate synthase; G3P, glyceraldehyde 3-phosphate; GA, gibberellin; GGPP, geranylgeranyl diphosphate; GGPPS, geranylgeranyldiphosphate synthase; GPP, geranyl diphosphate; GPPS, geranyl diphosphate synthase; HDR, 4-hydroxy-3-methylbut-2-enyl diphosphate reductase; HDS,4-hydroxy-3-methylbut-2-enyl diphosphate synthase; HMBPP, 4-hydroxy-3-methylbut-2-enyl diphosphate; HMG-CoA, 3-Hydroxy-3-methylglutary l-CoA; HMGR, hydroxymethylglutaryl-CoA reductase; HMGS, hydroxymethylglutaryl-CoA synthase; IDI, isopentenyl diphosphate isomerase; IDS, isoprenyl diphosphate synthase; IPP, isopentenyl diphosphate; KS, kaurene synthase; KSL, kaurene synthase-like; MCT, 2-C-methyl-D-erythritol 4-phosphate cytidylyltransferase; MDC, mevalonatepyrophosphate decarboxylase; MDS, 2-C-methyl-D-erythritol 2,4-cyclodiphosphate synthase; ME-cPP, 2-C-methyl-D-erythritol 2,4-cyclodiphosphate; MeJA, methyljasmonate; MEP, 2-C-methyl-D-erythritol 4-phosphate; MK, mevalonate kinase; MVA, mevalonate; MVAP, mevalonate-5-phosphate; MVAPP, mevalonate-5-diphosphate; PMK, 5-phosphomevalonate kinase; CYP76AH1, cytochrome P450 monooxygenase 76AH1. Red indicated enhanced expression or production; blue indicated repressed expression or production; white indicated the expression of control; darker color indicated more significant change. All the values were ratios of average gene expression level or compounds content in *MYC2-RNAi* hairy roots to control.

To further explore whether the biosynthesis of tanshinones and Sal B was regulated by *Sm*MYC2a and *Sm*MYC2b, RNAi-mediated expression knockdown experiments were carried out in *S. miltiorrhiza*. The RNAi silencing vectors *p1300-pHANNIBAL-MYC2a/b* recombinants (Supplementary Fig. S2A) were transformed into *Agrobacterium tumefaciens* C58C1 strain and subsequently introduced into *S. miltiorrhiza* leaf explants. Growing status of fresh hairy roots from leaf explant which carried *pCAMBIA1300-pHANNBIAL* vector was displayed in Supplementary Fig. S2B–D. PCR testing positive transgenic genes were harvested (Supplementary Fig. S2E,F). The expression of *SmMYC2a* and *SmMYC2b* decreased to 24.9% and 61.5% respectively of control in corresponding *MYC2-RNAi* hairy roots on average. In transgenic *SmMYC2a-RNAi* hairy roots, the expression of pathway genes was reduced, such as 3-hydroxy-3-methylglutaryl-CoA reductase (*HMGR*), mevalonate kinase (*MK*), 4-(cytidine 5’-diphospho)-2-C-methyl- D-erythritol kinase (*CMK*), 2C-methyl-D-erythritol 2,4-cyclodiphosphate synthase (*MCS*), geranyl diphosphate synthase (*GPPS*), geranylgeranyl diphosphate synthase (*GGPPS*), copalyl diphosphate synthase (*CPS*) and kaurene synthase-like protein (*KSL*) which were involved in the biosynthesis of tanshinones (Figs 5 and 6). Expression level of phenolic acid biosynthetic genes as phenylalanine ammonia-lyase (*PAL*), *HCT6*, 4-hydroxyphenylpyruvate reductase (*HPPR*) and *CYP98A14* also decreased in *SmMYC2a* knockdown lines. In contrast to *SmMYC2a* knockdown effect, *Sm*MYC2b not only affected several common genes (*SmPAL*, *SmHPPR*, *SmHCT6*, *SmCYP98A14*, *SmMK*, *SmCMK*, *SmMCS, SmCPS* and *SmKSL*), but also reduced the expression of *SmDXR*. The expression of *SmMYC2a/b* and pathway genes in control hairy roots and transgenic *Sm*MYC2a/b suppressed hairy roots were characterized in Supplementary Fig. S3 in a form of histogram.

### Accumulation of tanshinones and salvianolic acids was affected by RNAi silencing of *SmMYC2a* or *SmMYC2b*

To assess the effects of reduced *SmMYC2a* and *SmMYC2b* expression on tanshinones and phenolic acids biosynthetic gene expression, 10 compounds involved in tanshinones and Sal B synthetic pathways were determined and the corresponding chromatogram map was shown in Supplementary Fig. S4. Three *SmMYC2a* and four *SmMYC2b* knockdown lines with significant reduction of *MYC2a* or *MYC2b* were examined for their effects on production of tanshinone and phenolic acids. The ratios of the gene expression level or compounds content in *MYC2-RNAi* hairy roots to control were displayed in Figs 5 and 6. A significant decrease in the biosynthesis of phenolic acids (Sal A and Sal B) and intermediate products (L-phenylalanine, homogentisic acid, rosmarinic acid) was observed in *SmMYC2a* or *SmMYC2b* knockdown lines, showing a consistent repression effect. Moreover, both *SmMYC2a* and *SmMYC2b* knockdown lines presented an extremely low yield of tanshinone I, dihydrotanshinone I, tanshinone IIA, cryptotanshinone. The content of tanshinone IIA reduced to 6.4% and Sal B dropped to 17.2% compared with control lines on average in *SmMYC2a* knockdown hairy roots. The silencing of *SmMYC2b* caused similar changes in tanshinones accumulation with the production of tanshinone IIA reduced to 45.2% and Sal B dropped to 5.0% compared with control lines on average. Determination of compounds in transgenic *Sm*MYC2a/b knockdown hairy roots was characterized in Supplementary Fig. S5 in a form of histogram. These results indicated that *Sm*MYC2a and *Sm*MYC2b were positive regulators that affected the biosynthesis of tanshinones and Sal B.

### *Sm*MYC2a and *Sm*MYC2b could bind with the E-Box within the *SmCYP98A14* or *SmHCT6* promoter *in vitro*

Previous studies indicated that G-box (5′-CACGTG-3′) fragment was the preferred core binding site of MYC2, followed by 5′-CACATG-3′ and 5′-CACGTT-3′ motifs^19^. Thus, the screening of candidate MYC2 activated genes took the preferential order into consideration. *SmCYP98A14*, namely *SmCYP98A78* was reported as a JA-responsive gene^20^. Pathway genes as *SmCPS1* and *SmCYP76AH1* were previously investigated and proven to be JA inducible^21^,^22^. *SmHCT6* was also illustrated as a JA-inducible gene. The above four genes were discovered to contain E-box motifs in their promoters. Thus, they were selected as candidates of MYC2 activated genes.

**Figure 7:**
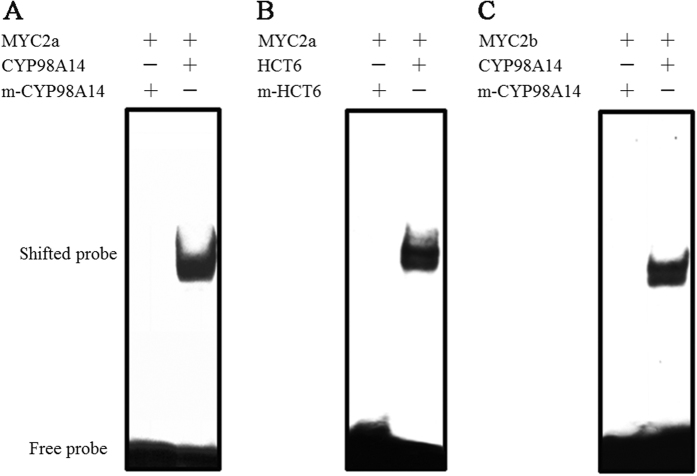
Specific binding of *Sm*MYC2a or *Sm*MYC2b to the E-Box fragments of the *SmHCT6* and *SmCYP98A14* promoter. (**A**,**B**) Specific binding of *Sm*MYC2a to E-box in *SmCYP98A14* and *SmHCT6* promoter. (**C**) Specific binding of *Sm*MYC2b to E-box in *SmCYP98A14* promoter.

Purified fusion proteins consisting of *Sm*MYC2a/b protein and cleavable His-tags were prepared (Supplementary Fig. S6). Promoters of pathway genes (*SmCYP98A14*, *SmHCT6*, *SmCPS1* and *SmCYP76AH1*) which contained E-box element were potential binding sites of MYC2. The promoters of the candidate genes were amplified by genomic walking and biotin-modified for EMS assay. EMS assays were performed using purified target proteins and biotin-modified promoter fragments containing the E-box sequences to test binding behavior. As presented in Fig. 7, C-terminally His-tagged *SmMYC2a* protein (named as *MYC2a-His* protein) bound to the *HCT6* oligonucleotide probe with intact E-box motif (lane 2), but was not capable of binding to promoter fragments in which the whole E-box was replaced by T residues (lane 1). Both MYC2a-His and MYC2b-His (C-terminally His-tagged *SmMYC2b*) proteins bound to G-box motif-containing *SmCYP98A14* promoter.

## Discussion

MeJA is widely used as an elicitor to investigate the biosynthetic pathway of active compounds and underlying regulatory mechanisms^14^. Previous research indicated that MeJA treatment promoted the accumulation of secondary metabolites, tanshinones and phenolic acids^15^,^16^,^17^,^23^. MYC2 is a master regulator of JA signaling pathway which plays a vital role in the activation of pathway genes in various model plants^19^,^24^,^25^. Whether MYC2 regulates the biosynthesis of tanshinones and phenolic acids still remains unknown yet. How MYC2 fine-tunes the pathway hasn’t been explored before in *S. miltiorrhiza*. In this study, two bHLH TFs, *SmMYC2a* and *SmMYC2b* were obtained from *S. miltiorrhiza.* Phylogenetic analysis result revealed that *Sm*MYC2a and *Sm*MYC2b showed high homologs to MYC2 proteins of many other species. Previous studies indicated that MYC2 was a master regulator with extensive regulating effect in JA signaling pathway. Among the well studied MYC2s, *At*MYC2 was reported as a regulator of glucosinolate (GS) biosynthesis, insect performance and feeding behavior^26^. *Nt*MYC2a and *Nt*MYC2b regulated multiple jasmonate-inducible steps in nicotine biosynthesis^27^.

Previous investigations demonstrated that JAZ repressors targeted bHLH TFs to control JA-dependent anthocyanin accumulation and trichome initiation^28^. As the *Sm*MYC2a and *Sm*MYC2b contained conserved JID, we reasoned that a similar complex might be formed to control the accumulation of tanshinones and phenolic acids. Y2H assay was implemented to investigate the interactive ability of *Sm*MYC2 with *Sm*JAZ repressors. The binding of the two proteins could enable the growth of host AH109 yeast strain on quadruple selection medium and activate the expression of MEL1 reporter gene, making α-gal turn blue. Both *Sm*MYC2s exhibited interactions with *Sm*JAZ1 and *Sm*JAZ2, implying that the two MYC2 TFs might function as direct targets of JAZ proteins. The binding effect of MYC2-JAZ was in accordance with previous findings in *Arabidopsis*^26^, which might result in the repression of MYC2 proteins that could bind to the promoters of critical pathway genes and activate their transcription. Both *SmMYC2a* and *SmMYC2b* were discovered to be JA-inducible, reaching a maximal level at 0.5 h after MeJA treatment. The early response pattern was in accordance with that in *N. benthamiana* and the maximum response occurred at 0.5 h^29^. Afterwards, the variation trends of transcript levels of *SmMYC2a* and *SmMYC2b* slightly differed. Transcript level of *SmMYC2a* declined slowly till 3 h, which was similar to changes of *NaMYC2*. However, transcript level of *SmMYC2b* decreased sharply till 1 h after MeJA treatment. The different reduction velocity of *SmMYC2a* and *SmMYC2b* transcripts indicated that compared to *SmMYC2b*, *SmMYC2a* could maintain a high expression longer and might serve as a regulator with more extensive modulatory effects. Our results also demonstrated that *SmHCT6* and *SmCYP98A14* were remarkable JA-responsive genes and the response occurred relatively early. The JA-mediated regulation profile of positive pathway genes was in parallel with that in *N. benthamiana*^29^.

After silencing *SmMYC2a* or *SmMYC2b*, qRT-PCR analysis was performed to seek for evidence of the relationships between the two TFs and critical pathway genes. Not only the phenolic acids biosynthetic genes were affected, but also the tanshinone pathway was repressed in *SmMYC2a* knockdown lines. Most of the pathway genes influenced by *Sm*MYC2a had been previously studied. *SmCMK* helped to enhance the accumulation of tanshinones^30^. *SmHMGR* and *SmDXR* were critical pathway genes whose overexpression could significantly enhance the yield of tanshinone in hairy root lines, while co-expressing produced evidently higher levels of total tanshinone, which provide a useful strategy to improve tanshinone content^31^. *SmGGPPS* played a more important role in stimulating tanshinone accumulation than the upstream enzyme *SmHMGR*^32^. The decreased expression of *SmCPS* caused a decrease in tanshinone levels which confirmed *SmCPS* as a key enzyme for tanshinone biosynthesis in *S. miltiorrhiza*^33^. Rosmarinic acid synthase (RAS), namely HCT also played a vital role in the biosynthesis of Sal B. In *SmRAS* antisense transgenic *S. miltiorrhiza* hairy root lines, the accumulation of RA and Sal B obviously decreased to 59% and 42% of control on average, and in *CYP98A14*-antisense transgenic lines, the content of RA and Sal B decreased to 44% and 81% of control on average^34^. In *MYC2-RNAi* hairy roots, several genes were found to be dramatically reduced, including *SmPAL*, *SmHPPR*, *SmHCT6*, *SmCYP98A14*, *SmMK*, *SmCMK*, *SmMCS*, *SmCPS* and *SmKSL*. Additionally, the expression of *SmDXR* decreased only in *SmMYC2b* knockdown hairy roots, while *SmHMGR*, *SmGPPS* and *SmGGPPS* were found to be repressed in *SmMYC2a* knockdown hairy roots, indicating *Sm*MYC2a and *Sm*MYC2b were positive regulators that strongly regulated pathway genes in the biosynthesis of tanshinones and phenolic acids. To assess the effects of reducing *SmMYC2a*/*b* expression on tanshinone and phenolic acid biosynthesis, all the PCR-tested positive hairy roots were subjected to compounds analysis. A significant decrease was observed in the biosynthesis of intermediate product (L-phenylalanine, homogentisic acid, rosmarinic acid) and end products (Sal A, Sal B) in *SmMYC2a* or *SmMYC2b* knockdown lines, showing a consistent repression effect of phenolic acid biosynthesis pathway. The biosynthesis of tanshinones (tanshinone I, dihydrotanshinone I, tanshinone IIA, cryptotanshinone) was remarkably depressed in *SmMYC2a* knockdown transgenic lines, and knockdown of *SmMYC2b* caused similar changes in the production of tanshinones. The positive regulating effect of MYC2 was in parallel with previous studies on *Arabidopsis* and *Artemisia annua*^19^,^25^,^35^.

To further explore the genes that MYC2 could transcriptionally activate, EMS assays were performed using purified target proteins and biotin-modified promoter fragments containing the E-box. Purified *Sm*MYC2a fusion protein was discovered to bind to the E-box containing *SmHCT6* and *SmCYP98A14* oligonucleotide probes. *Sm*MYC2b fusion protein could bind to E-box motif in *SmCYP98A14* promoter. The binding effect revealed that *Sm*MYC2a might regulate the biosynthesis pathway of phenolic acids by activating the transcription of *SmHCT6* and *SmCYP98A14* while the regulation of *Sm*MYC2b might be via the binding to *SmCYP98A14.*

**Figure 8:**
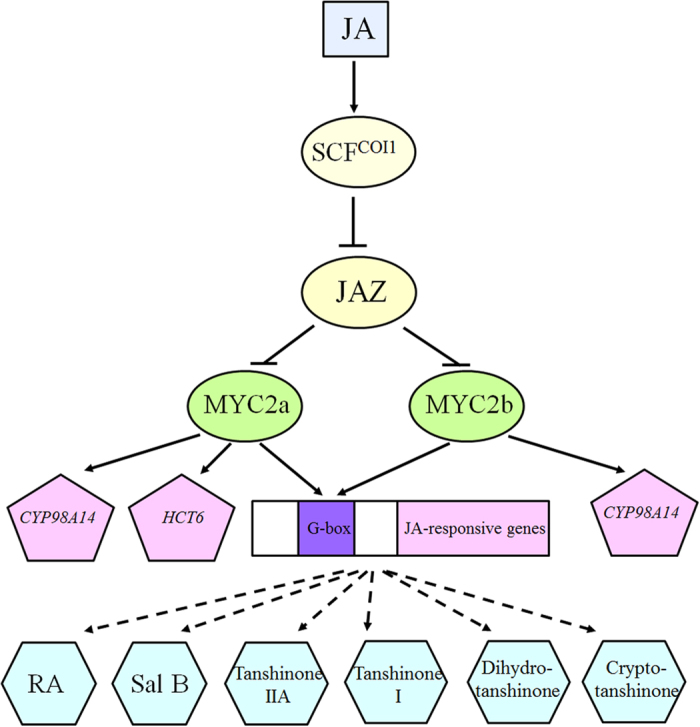
Schematic model of the biosynthesis regulation of tanshinones and phenolic acids by *Sm*MYC2a and *Sm*MYC2b.

Our current understanding of biosynthetic regulation of tanshonone and phenolic acid pathway by MYC2 was illustrated in Fig. 8. In the presence of bioactive jasmonate stress, JA was conjugated to isoleucine to form JA-Ile and subsequently bound SCF^COI1^ complex and JAZ proteins, leading to the degradation of JAZ proteins and the release of MYC2 TFs. *Sm*MYC2a might regulate the biosynthesis pathway of phenolic acids by activating the transcription of *SmHCT6* and *SmCYP98A14* while the regulation of *Sm*MYC2b might be via the binding to *SmCYP98A14.* JA-MYC2-genes-active compounds network was roughly constructed. However, it is still far away from fully understanding for regulating role of MYC2 in secondary metabolites. More attention should be paid on the characterization of MYC2 TFs in JA pathway to provide additional evidence for the extensive regulating effect and help to better understand the complex JA regulatory network.

In conclusion, our results indicate that *Sm*MYC2a and *Sm*MYC2b are positive regulators which exert similar but also irreplaceable functions in the regulation of JA-responsive genes, leading to the biosynthetic regulation of tanshinones and phenolic acids. The functional characterization of *Sm*MYC2a and *Sm*MYC2b which regulate multiple jasmonate-inducible steps in the biosynthesis of active compounds in *S. miltiorrhiza* will definitely facilitate better understanding of regulating mechanism of JAs on active compounds. As *Sm*MYC2a and *Sm*MYC2b are positive regulators which affect a wide array of pathway genes, high yield of target compounds in *S. miltiorrhiza* may be achieved by metabolic engineering strategy using *Sm*MYC2a and *Sm*MYC2b as candidates.

## Materials and Methods

### Plant growth and hairy root cultures

*S. miltiorrhiza* plants were grown in the botanical garden of the Second Military Medical University and identified by Professor Hanming Zhang. Fresh roots, stems and leaves were separated from 2-month-old *S. miltiorrhiza* seedling, washed with distilled water, dried with filter paper and stored immediately at −70 °C for organ expression analysis. To obtain sterilized seedlings, seeds of *S. miltiorrhiza* were thoroughly sterilized utilizing 75% ethanol for 5 min and subsequent 1% HgCl_2_ solution for 10 min. After rinsed thoroughly with sterile distilled water for 5 times, the seeds were sown on prepared Murashige and Skoog (MS) medium (Sigma-Aldrich, St Louis, MO, USA) plus 3% sucrose and 0.5% agar with the pH value adjusted to 5.6.

Hairy roots of *S. miltiorrhiza* were derived from plantlets infected with *Agrobacterium rhizogenes* bacterium of C58C1 strain and maintained subculturing after sterilization. MeJA treatment assay was performed on *S. miltiorrhiza* hairy roots which were shaking cultured and reached exponential growth phase. Dissolved in ethanol, MeJA was added to a final concentration of 50 μM into *S. miltiorrhiza* hairy roots medium. Hairy roots from MeJA-treated group and solvent control group were harvested at selected times (0, 0.5, 1, 2, 4, 6, 12 and 24 h) and stored at −70 °C for gene expression analysis^27^.

Subcellular localization experiment was performed on rice protoplast which was derived from rice seedlings. Rice seeds were sterilized in 75% ethanol for 1 min and then disposed with 33% sodium hypochlorite solution for 5 min. After thoroughly sterilized using 0.1% HgCl_2_, the seeds were rinsed thoroughly with sterile distilled water for 5 times and placed on MS medium plus 0.5% sucrose and 0.5% agar with the pH value adjusted to 5.8. After culturing under light at 25 °C for 3 days, the height of shoots reached 1 cm. Then, the rice seedlings were placed in a dark environment and used for protoplast separation after 14-day-culture in dark.

### Cloning and phylogenic analysis of *SmMYC2a* and *SmMYC2b*

Based on transcription profiling database previously established^18^, we amplified *SmMYC2a* with primers 5′-ATGATTGATTACCGCACGCC-3′ and 5′-CTATCTAATCTCAGCAACTTTAG-3′, and *SmMYC2b* with 5′-ATGGGGGTTGTTGGTTGG-3′ and 5′-TTACCCGAGAGATAACTGATG-3′ using total RNA from *S. miltiorrhiza* seedling as amplification template. The two novel *SmMYC2* genes were used to create multiple sequence alignments with *AtMYC2* (GenBank accession: NM_102998.3) on ClustalX 2 at amino acid level^36^. To establish the evolutionary relationships between *Sm*MYC2s and their best homologs, amino acid sequences of *Sm*MYC2a and *Sm*MYC2b were used as query sequences to search for homologs in NCBI (http://www.ncbi.nlm.nih.gov/) using BLAST. The two *Sm*MYC2s, together with 37 closest MYC2s from other species (Supplementary Table S1), were used to construct a Neighbour-joining (NJ) tree using MEGA 5.05 (http://www.megasoftware.net) with 1000 bootstrap replicates using full-length amino acid sequences.

### Vector construction and plant transformation

DNA fragment of GUS in *pCAMBIA1301* vector was changed into GFP to get the modified *pCAMBIA1301-GFP* vector which could serve as a subcellular localization vector. cDNA of *SmMYC2* without termination codon was inverted into *pCAMBIA1301-GFP* vector to get *pCAMBIA1301-GFP-SmMYC2* which expressed corresponding recombinant protein.To construct RNAi silencing vectors of *SmMYC2*, a 400-bp DNA fragment circumventing conserved bHLH domain was amplified. The modified *pCAMBIA1300-pHANNBIAL* vector which contained a 35S promoter, a PDK intron and an OCS terminal was used as the RNAi vector. The amplified 400-bp *SmMYC2* fragment was forwardly and reversely inverted to the two ends of the PDK intron to get *pCAMBIA1300-pHANNBIAL-MYC2* vector. The constructed vectors were separately introduced into the disarmed *Agrobacterium tumefaciens* C58C1 strain which carried the rooting plasmid pRiA4^37^. The *pCAMBIA1300-pHANNBIAL* vector was also transformed as control using the same method. Modified PHB-FLAG vector was constructed by introducing the FLAG tag and *Spe* I restriction site into the MCS region with primers *Bam* HI and *Sac* I. The complete coding region of *SmMYC2a/b* was amplified and inserted into the modified binary vector *PHB-Flag*. The final construct was transferred into *Agrobacterium tumefaciens* (C58C1 strain) and introduced into leaf explant from *S*. *miltiorrhiza*. The plants which carried *PHB-Flag* vector were used as control. The prokaryotic expression vectors which expressed *SmMYC2a-His* and *SmMYC2b-His* proteins were constructed by introducing the whole length-amplified *SmMYC2a* or *SmMYC2b* into *pET32a* vector using *Bam* HI and *Hind* III, *Eco* RV and *Sal* I restriction sites. Yeast two-hybrid assay was applied to investigate the interaction of *SmJAZ1/2* and *SmMYC2a/b in vitro*. The whole coding sequences of *SmJAZ1/2* and *SmMYC2a/b* were introduced into *pGADT7* vectors between *Nde* I and *Eco* RI*, Nde* I and *Xma* I restriction sites while *SmJAZ1/2* were inserted into *pGBKT7* vectors between *Nde* I and *Eco* RI*, Nde* I and *Xma* I restriction sites, respectively. All the primers used in the construction of vectors were shown in Supplementary Table S2.

### Subcellular localization of *Sm*MYC2a and *Sm*MYC2b

Transient expression assay was performed to explore subcellular localization. The *pCAMBIA1301-GFP-MYC2* vector which expressed GFP-tagged fusion protein was transformed into freshly prepared rice protoplast using PEG transfection method^38^. Subcellular localizations of GFP, MYC2a-GFP, and MYC2b-GFP proteins were determined by microscopy assay with a fluorescence microscopy. GFP fluorescent signals were visualized with an excitation wave length of 488 nm and an emission wavelength of 505–530 nm. The red autofluorescence resulting from chlorophylls was captured by an emission wavelength longer than 650 nm. The processing of captured images was conducted using Photoshop software.

### Yeast two-hybrid assay

Y2H assay was conducted to detect the interaction of JAZ with bHLH TF *in vitro*. Recombinant vector of *pGBKT7-JAZ1/2* that contained GAL4 DNA-binding domain served as a bait protein, and *pGADT7-MYC2a/b* vector that contained GAL4 activation domain acted as a prey protein. The two types of recombinant vectors were co-transformed into yeast strain AH109 using the PEG/LiAC method. Empty vectors of *pGADT7* and *pGBKT7* were also co-transformed as a negative control. After selection on synthetic SD dropout medium lacking leucine and tryptophan (SD-Leu-Trp), single transformant colonies were then screened for growth on SD selection medium lacking adenine, histidine, leucine and tryptophan (SD-Ade-His-Leu-Trp). α-gal was dissolved in DMF to a concentration of 4 mg/ml and then smeared uniformly on quadruple SD medium. Transformant colonies that turned blue indicated the interactions of *Sm*MYC2a/b and *Sm*JAZ1/2. To test potential auto-activation of the preys, vectors of *pGADT7-MYC2a/b* and *pGBKT7* were also co-transformed as controls.

### Gene expression analysis

Total RNA was isolated using a total RNA Isolation Kit (TIANGEN BIOTECH (BEIJING) CO., LTD, Beijing, China) and DNA contamination was eliminated by DNase treatment. After measuring quality and concentration of RNA obtained, 1μg of total RNA was reverse-transcribed to obtain cDNA using TransScript First-Strand cDNA Synthesis SuperMix Kit (TransGen Biotech, Beijing, China) according to manufacturer’s instructions. Quantitative RT-PCR (qRT-PCR) was performed following the instructions of SYBR^®^PreMix Ex Taq (Takara Bio Inc., Dalian, China) on Thermal Cycler Dice Real Time System TP800 (Takara Bio Inc., Dalian, China), with primers listed in Supplementary Table S3. Efficiency-corrected comparative Ct method was applied and relative expression was calculated by normalizing interested genes according to the abundance of the housekeeping gene (18S ribosomal subunit, namely *Sm*Actin). All qRT-PCR experiments were performed with three independent replicates. Expression levels of *SmMYC2a*, *SmMYC2b* and main pathway genes were determined by qRT-PCR.

### Compounds extraction and analysis

The hygromycin selected hairy roots were cultured in liquid medium for 45 days. Afterwards, positive transgenic strains were confirmed by PCR testing with primers listed in Supplementary Table S4. All the independent control clones and typical transgenic hairy roots were subjected to gene and compound analysis. The hairy roots were dried at room temperature for 2 days to a constant dry weight and milled to a homogeneous size of which No. 100 mesh could be sieved through. Active compounds (tanshinones and Sal B) were extracted according to the reported method^16^,^39^. 0.02 g powder sample was extracted in 70% methanol under sonication for 30 min and then centrifuged at 10,000 rpm for 5 min. The extraction volume was 5 mL and the weight loss was made up with 70% methanol after extraction. The extracted solution was filtered through a 0.22 μm organic membrane before determination.

A method of performance liquid chromatography-tandem mass spectrometry (LC-MS/MS) has been developed for the determination analysis of 10 compounds in *S. miltiorrhiza* hairy root cultures. The separation of lipophilic tanshinones and hydrophilic phenolic acids was performed by a common Waters xSELECT CSH^TM^ C18 column (2.1 mM × 50 mM, 2.5 μM, Agilent Corporation, MA USA) in two ion monitoring modes. When analyzing 6 hydrophilic phenolic acid compounds, we selected the gradient elution approach by using a mobile phase acetonitrile (A) - H_2_O (B) (containing 2 mmol/L ammonium acetate and 0.1% formic acid) 0 min (7% A) →1 min (7% A) →2.5 min (93% A) →7 min (93% A), and the flow rate was 0.3 mL/min in the progress. For lipophilic tanshinone analysis, an isocratic mobile phase consisting of acetonitrile (A) - H_2_O (B) (containing 2 mmol/L ammonium acetate and 0.1% formic acid) (60:40) was applied with a flow rate of 0.3 mL/min. The column temperature was 35 °C and the sample injection volume was 10 μL. The components were detected by an Agilent G6410A triple quadrupole LC/MS system equipped with a Mass hunter interface. The 10 hydrophilic phenolic acid compounds were detected using ESI in negative-ion mode, and the lipophilic tanshinones in positive-ion mode, quantified by multiple-reaction monitoring (MRM) mode using following transition mass of m/z, 164→147 for L-phenylalanine, 167→123 for homogentisic acid, 163→119 for 4-coumaric acid, 359.1→161.1 for rosmarinic acid (RA), 493→294.9 for Sal A, 717.1→519.1 for Sal B, 297.2→254.0 for cryptotanshinone, 277.1→249.1 for tanshinone I, 295.1→276.8 for tanshinone IIA, 279.1→233.1 for Dihydrotanshinone I. All the standards were purchased from Sigma-Aldrich (St. Louis, MO). Data analysis was carried out using the Agilent Mass Hunter Workstation software.

### Electronic mobility shift assay

The PCR-amplified cDNA fragment of *SmMYC2* was inserted into *pET32a* vector, and the constructed plasmid *pET32a-MYC2* was introduced into *E. coli* strain BL21 (DE3). Transformants were cultured at 37 °C until OD 600 reached about 0.5. Afterwards, to induce the expression of target protein, isopropyl thiob-D-galactoside (IPTG) was added to a final concentration of 1 mM. Further incubation was conducted at 18 °C for 2 days. After ultrasonic cell disruption, target proteins were purified based on immobilized metal affinity chromatography technique according to manufacturer’s instruction (Bio-Rad Laboratories, Hercules, CA). E-box elements, especially G-box were universally known as well-characterized binding sites of bHLH TFs which mediated JA responsiveness in *Arabidopsis*^19^,^40^. As genomic regions flanking E-Box binding sites influence DNA binding specificity of bHLH transcription factors through DNA shape^41^, DNA fragments of 30 bp length which contained the preferred binding site and flanking genomic regions were designed to detect protein-nucleic acid interactions in EMSA.

Wild-type HCT6: 5′-AAGCCTAAAGCTCACATGGCTGCTAAACCT-3′. m-HCT6 (G-box mutation in HCT6 promoter): 5′-AAGCCTAAAGCTTTTTTTGCTGCTAAACCT-3′. Wild-type CYP98A14: 5′-ATTAGTGATTAACACGTGCACATTTATTAA-3′. m-CYP98A14 (G-box mutation in CYP98A14 promoter): 5′-ATTAGTGATTAATTTTTTCACATTTATTAA-3′.

To detect protein-nucleic acid interactions *in vitro*, EMS assay was performed. 2 μg of purified recombinant protein was incubated with 2 μg of 3′ biotin-labeled probes at room temperature for 10 min. After binding reaction, free nucleic acid was separated from the formed complexes by polyacrylamide gel electrophoresis. Subsequently, the gel was transferred to nylon membrane, crosslink transferred and chemiluminescence detected according to manufacturer’s protocol (TransGen Biotech, Beijing, China).

### Statistical analysis

Statistical analysis was conducted using SPSS 16.0 software (e.g. analysis of variance (ANOVA) for between-treatment assessment and Student’s t test for pair comparisons to determine significant differences between means).

## Additional Information

**How to cite this article**: Zhou, Y. *et al.* *Sm*MYC2a and *Sm*MYC2b played similar but irreplaceable roles in regulating the biosynthesis of tanshinones and phenolic acids in *Salvia miltiorrhiza*. *Sci. Rep.* **6**, 22852; doi: 10.1038/srep22852 (2016).

## Supplementary Material

Supplementary Information
