# Author Correction: *Sm*MYC2a and *Sm*MYC2b played similar but irreplaceable roles in regulating the biosynthesis of tanshinones and phenolic acids in *Salvia miltiorrhiza*

**DOI:** 10.1038/s41598-020-62994-w

**Published:** 2020-04-24

**Authors:** Yangyun Zhou, Wei Sun, Junfeng Chen, Hexin Tan, Ying Xiao, Qing Li, Qian Ji, Shouhong Gao, Li Chen, Shilin Chen, Lei Zhang, Wansheng Chen

**Affiliations:** 1 Department of Pharmacy, Changzheng Hospital, Second Military Medical University, Shanghai, 200003 China; 2 Institute of Chinese Materia Medica, China Academy of Chinese Medicinal Sciences, Beijing, 100700 China; 3 Department of Pharmaceutical Botany, School of Pharmacy, Second Military Medical University, Shanghai, 200433 China

Correction to: *Scientific Reports* 10.1038/srep22852, published online 07 March 2016

This Article contains an error in Figure 3, where the AD: MYC2a images for SD-L-W for both BD: JAZ1 and BD: JAZ2 are a duplication of the AD: MYC2a image for SD-L-W for BD: JAZ2. The correct Figure 3, which also contains higher resolution versions of the AD: MYC2b and AD images for SD-L-W for both BD: JAZ1 and BD: JAZ2, appears below as Figure 1.

**Figure 1:**
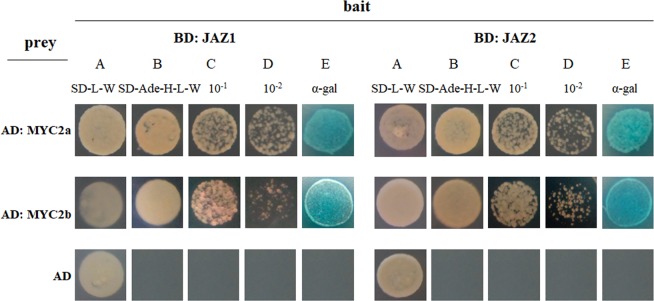
.

