# Supplementary material for: SmMYC2a and SmMYC2b played similar but irreplaceable roles in regulating the biosynthesis of tanshinones and phenolic acids in Salvia miltiorrhiza
Source: Sci Rep. 2016 Mar 7;6:22852. doi: 10.1038/srep22852 (PMC4780012; doi:10.1038/srep22852)
Supplement: Supplementary Information [file srep22852-s1.pdf]

_Sm_MYC2a and _Sm_MYC2b played similar but irreplaceable roles in regulating the biosynthesis of tanshinones and phenolic acids in _Salvia miltiorrhiza_

Yangyun Zhou[1], Wei Sun[2], Junfeng Chen[1], Hexin Tan[3], Ying Xiao[1], Qing Li[1], Qian Ji[3], Shouhong Gao[1], Li Chen[1], Shilin Chen[2], Lei Zhang[3*] and Wansheng Chen[1*]

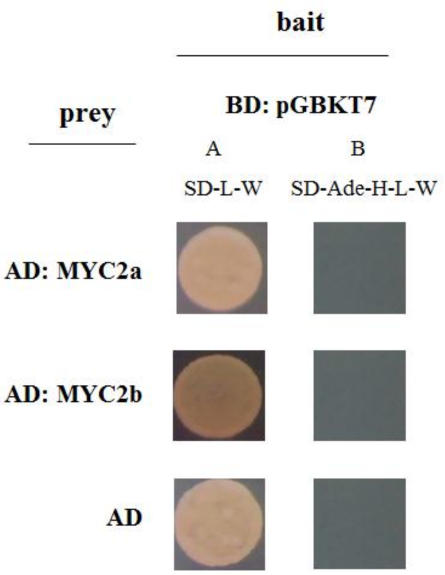

Supplementary Figure S1. *Sm*MYC2a and *Sm*MYC2b were not capable of auto-activating reporting genes in Y2H assay.

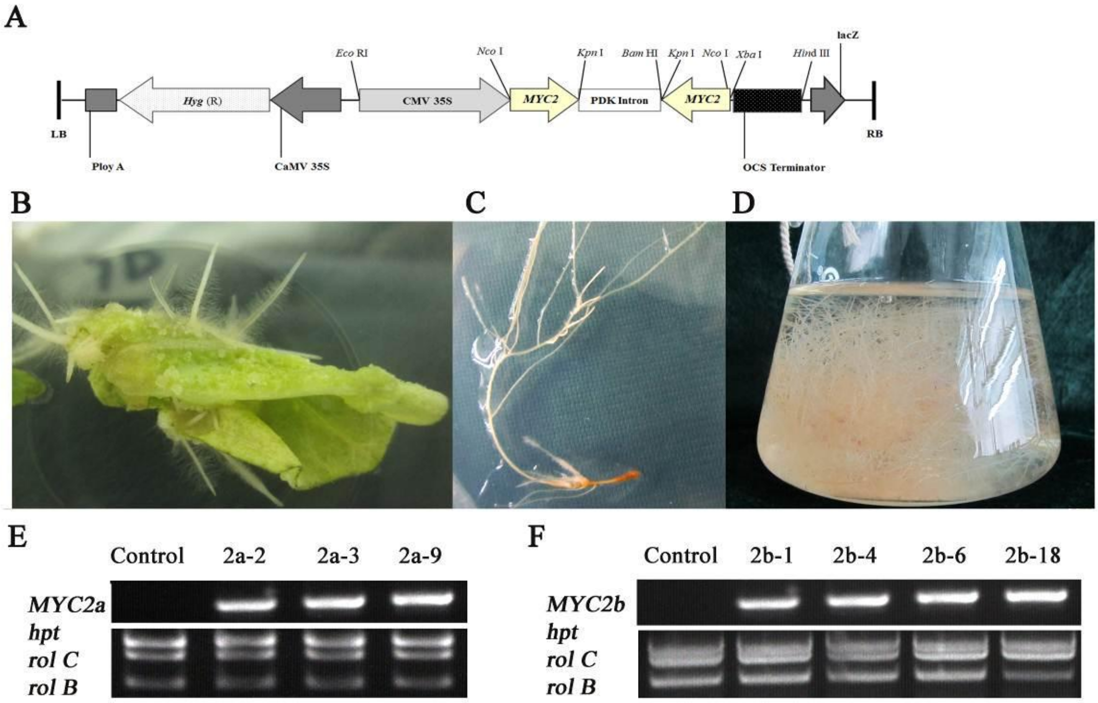

Supplementary Figure S2. Construction of *MYC2a/b-RNAi* transgenic hairy root lines.

(A) Schematic representation of *pCAMBIA1300-pHANNBIAL* vector.

(B) Fresh hairy roots from leaf explant which carried *pCAMBIA1300-pHANNBIAL* vector.

(C) Hairy roots of (B) after hygromycin selection.

(D) Hairy roots of (B) in liquid medium.

(E, F) Indentification of *MYC2a*/*b* transgenic hairy roots by PCR amplification of *MYC2*, *hpt*, *rol B*, and *rol C* genes.

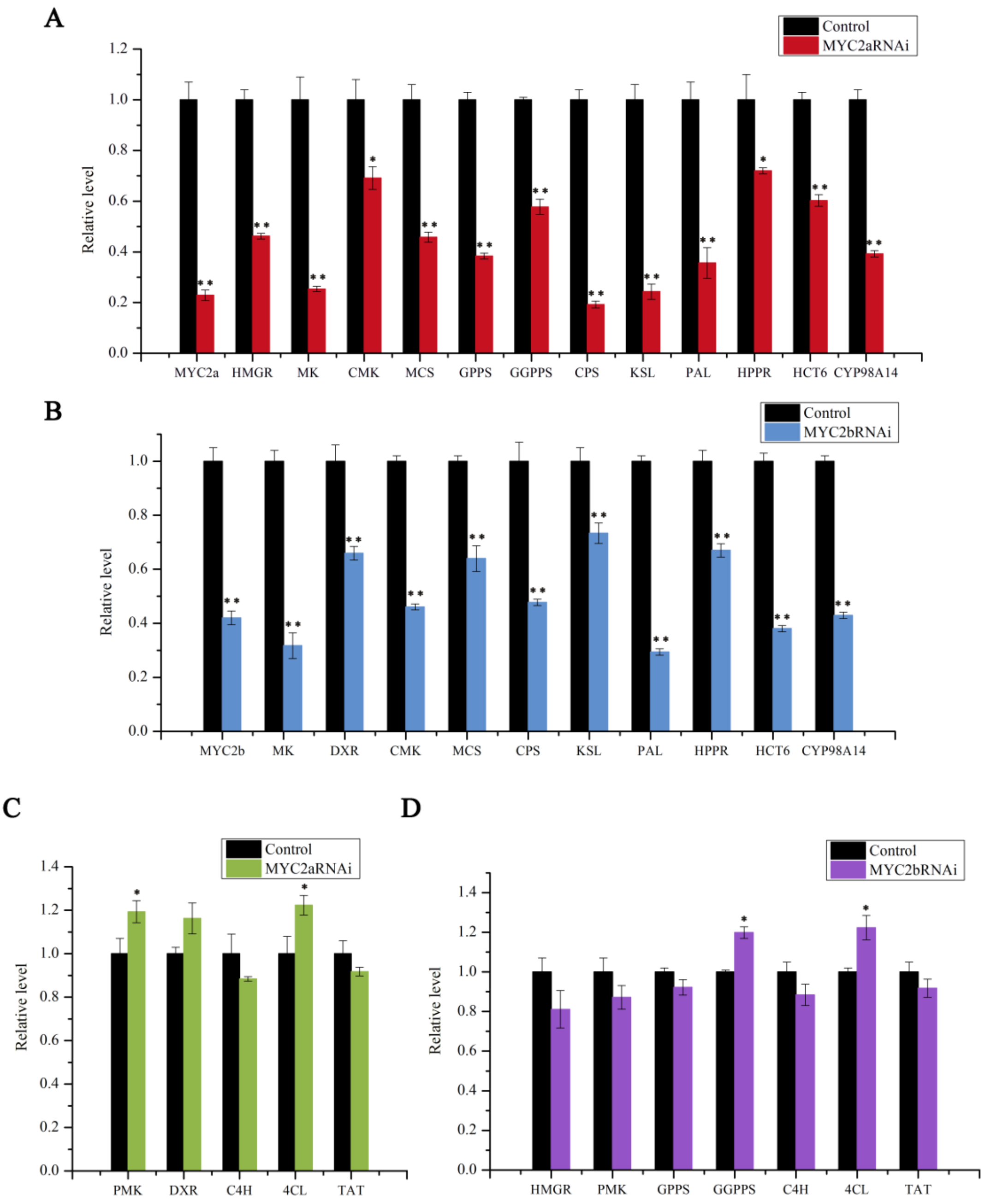

Supplementary Figure S3. Expression of *SmMYC2a/b* and pathway genes in control hairy roots and transgenic *Sm*MYC2a/b suppressed hairy roots. Each data point was the average of three or four biological replicates. *Actin* was used as a control for normalization. Error bars indicated SD. "*" indicated *p* <0.05 and "**" indicated *p* <0.01.

(A) Expression of significantly repressed genes in *MYC2a-RNAi* transgenic hairy roots.

(B) Expression of significantly repressed genes in *MYC2b-RNAi* transgenic hairy

roots.

(C, D) Other genes tested in *MYC2a/b-RNAi* transgenic hairy roots.

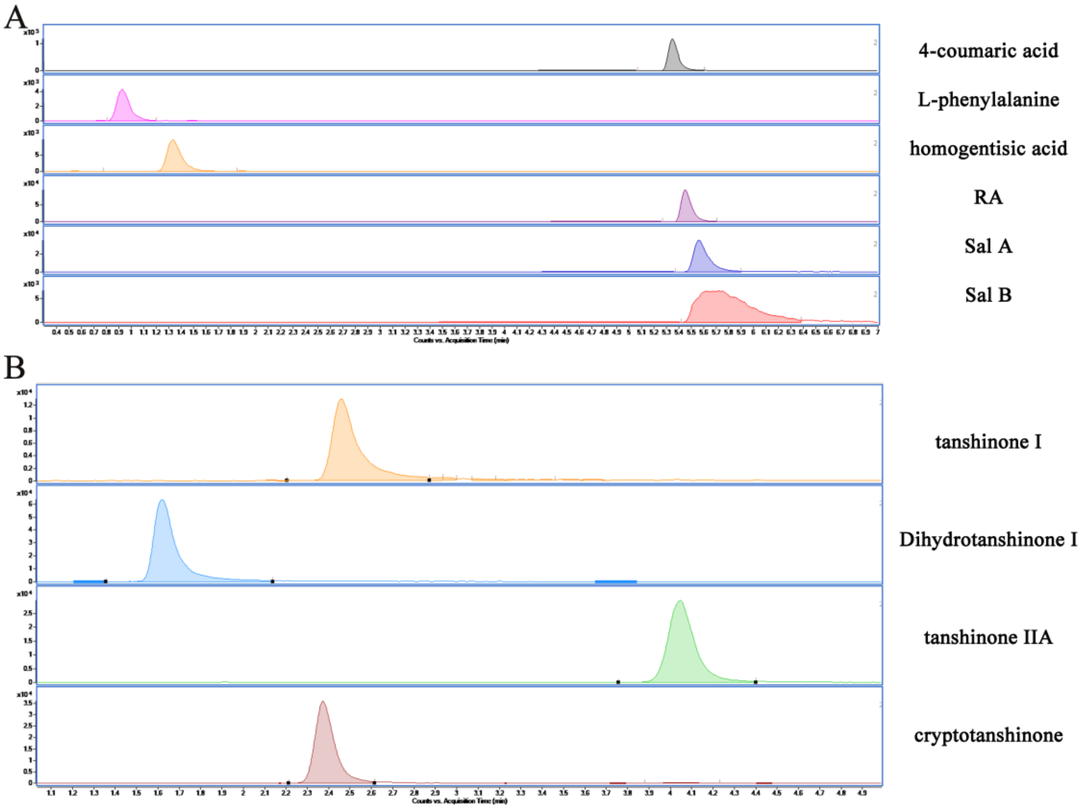

Supplementary Figure S4. Chromatogram map of 10 standard substances determined.

(A) 6 hydrophilic phenolic acid compounds using ESI in negative-ion mode.

(B) 4 lipophilic tanshinones using positive-ion mode.

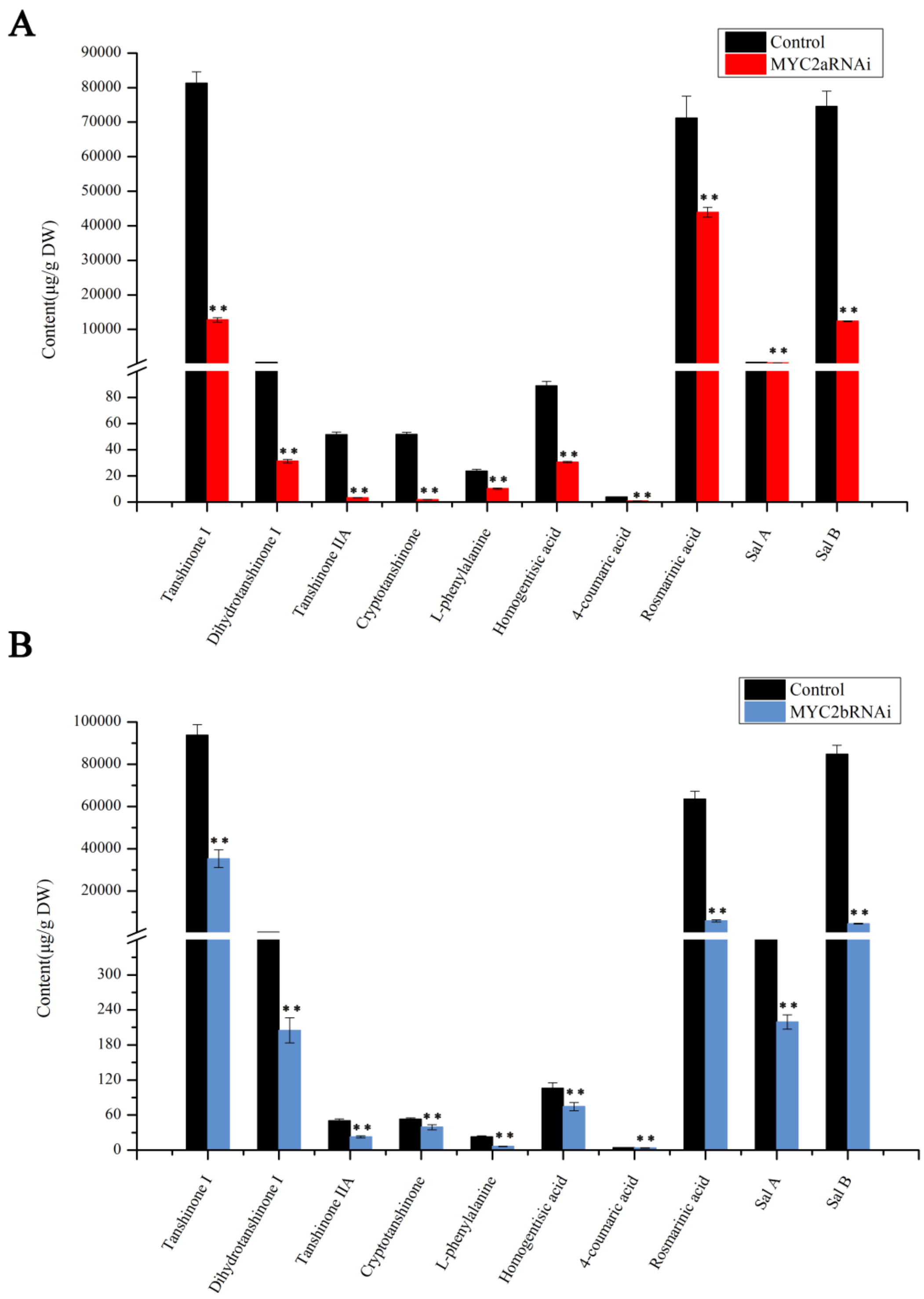

Supplementary Figure S5. Determination of content of target compounds in transgenic *MYC2a/b-RNAi* hairy roots. "*" indicated p <0.05 and "**" indicated p <0.01.

(A) The yield of compounds was affected by RNAi of *SmMYC2a*. Each data point was the average of three biological replicates. Error bars indicated SD.

(B) Content of compounds changed in transgenic *SmMYC2b* hairy roots compared with control group. Each data point was the average of three or four biological replicates. Error bars indicated SD.

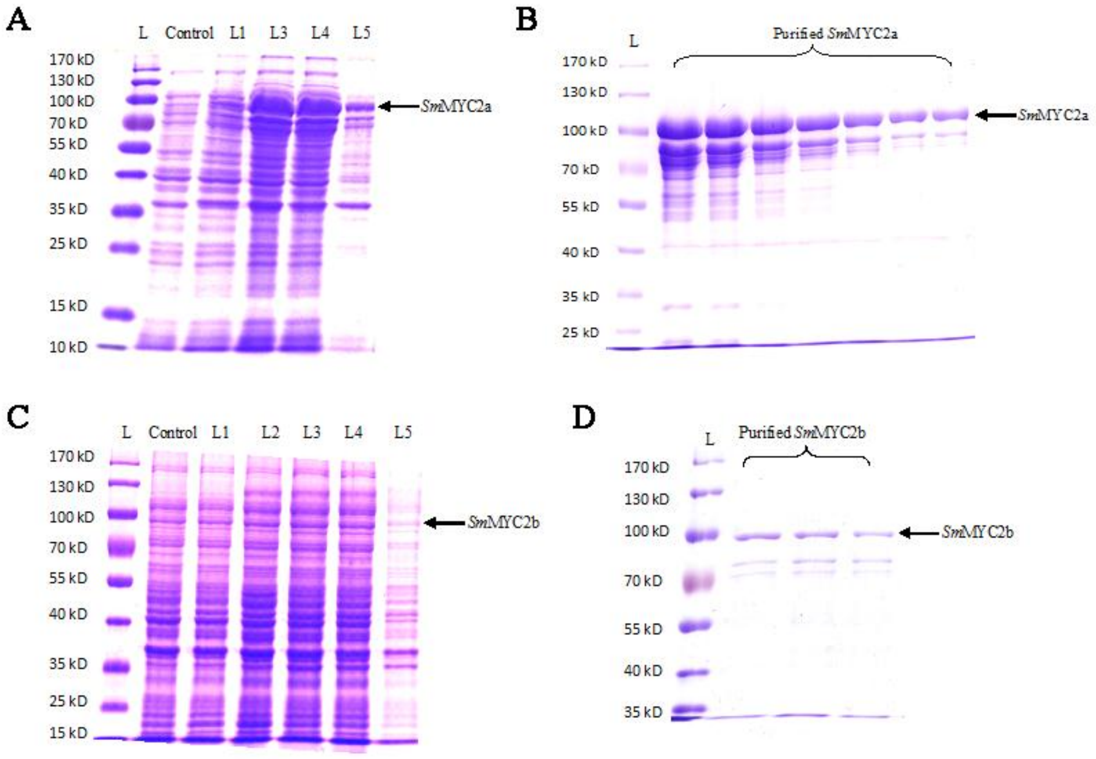

Supplementary Figure S6. Expression and purification of *Sm*MYC2a and *Sm*MYC2b proteins.

L: Prestained Protein Ladder (10-170 kD);

Control, uninduced *E.coli* strain BL21(DE3) which carried *pET-32a*;

Lane 1, induced *E.coli* strain BL21(DE3) which carried *pET-32a* by 1mM IPTG;

Lane 2, uninduced *E.coli* strain BL21(DE3) which carried *pET-32a-MYC2*;

Lane 3, induced *E.coli* strain BL21(DE3) which carried *pET-32a-MYC2* by 1mM IPTG;

Lane 4, supernatant of Lane 3;

Lane 5, precipitation of Lane 3.

(A, B) Recombinant *Sm*MYC2a protein expressed in *E.coli* BL21 (DE3) and purified *Sm*MYC2a protein applying immobilized metal affinity chromatography technique.

(C, D) SDS-PAGE analysis of recombinant and purified *Sm*MYC2b proteins.

Supplementary Table S1. Information of released bHLH genes used in the construction of phylogenetic tree.

Name	Gene ID	Plant	Family
AlbHLH	XM_002892031.1	*Arabidopsis lyrata* subsp. *lyrata*	Brassicaceae
AlbHLH2	XM_002882027.1	*Arabidopsis lyrata* subsp. *lyrata*	Brassicaceae
AlMYC2	XM_002893686.1	*Arabidopsis lyrata* subsp. *lyrata*	Brassicaceae
AtabHLH13	KD597917.1	*Aegilops tauschii*	Poaceae
AtbHLH13	NM_100009.2	*Arabidopsis thaliana*	Brassicaceae
AtbHLHai	NM_130216.2	*Arabidopsis thaliana*	Brassicaceae
AtMYC2	NM_102998.3	*Arabidopsis thaliana*	Brassicaceae
AtMYC4	NM_117897.3	*Arabidopsis thaliana*	Brassicaceae
BoMYC	EF423803.1	*Brassica oleracea* var. *gemmifera*	Brassicaceae
CsMYC2	AF283507.2	*Catharanthus roseus*	Vinceae
CsMYC2a	CM002924.1	*Cucumis sativus*	Vinceae
GhbHLH	FJ358540.1	*Gossypium hirsutum*	Malvaceae
GobHLH13	KN667611.1	*Glycine soja*	Fabaceae
GsbHLH13	KN660483.1	*Glycine soja*	Fabaceae
LeMYC1	KC818627.1	*Lithospermum erythrorhizon*	Boraginaceae
MtbHLH1	XM_003612861.1	*Medicago truncatula*	Fabaceae
MtbHLH2	CM001224.2	*Medicago truncatula*	Fabaceae
MtbHLH3	CM001223.2	*Medicago truncatula*	Fabaceae
MtbHLHai	CM001224.2	*Medicago truncatula*	Fabaceae

MtMYC2	XM_003628772.1	*Medicago truncatula*	Fabaceae
NaMYC2	KC832837.1	*Nicotiana attenuata*	Solanaceae
NaMYCl	KC906192.1	*Nicotiana attenuata*	Solanaceae
NbbHLH1	GQ859152.1	*Nicotiana benthamiana*	Solanaceae
NbbHLH2	GQ859153.1	*Nicotiana benthamiana*	Solanaceae
NtMYC1b	GQ859159.1	*Nicotiana tabacum*	Solanaceae
NtMYC2a	GQ859160.1	*Nicotiana tabacum*	Solanaceae
NtMYC2c	GQ859158.1	*Nicotiana tabacum*	Solanaceae
PsbHLH	KJ466971.1	*Paeonia suffruticosa*	Paeoniaceae
PtbHLH	XM_002302601.2	*Populus trichocarpa*	Salicaceae
RabHLH	DQ078120.1	*Rheum australe*	Polygonaceae
SlMYC2	KF428776.1	*Solanum lycopersicum*	Solanaceae
SmfMYC2	KJ139992.1	*Salvia miltiorrhiza f. alba*	Lamiaceae
StMYCa	AJ630505.1	*Solanum tuberosum*	Solanaceae
StMYCb	AJ630506.1	*Solanum tuberosum*	Solanaceae
TcbHLH	XM_007039431.1	*Theobroma cacao*	Malvaceae
TcMYC2i	XM_007051465.1	*Theobroma cacao*	Malvaceae
VvMYC2	NM_001281045.1	*Vitis vinifera*	Vitaceae

Supplementary Table S2. Primers used for vector construction.

Primer name	Sequence(5'→3')	Note
*SmMYC2a*sclF	AAAGATCTTATGATTGATTACCGCACGCC	Subcellecularlocalization (*SmMYC2a*)
*SmMYC2a*sclR	AAACTAGTTCTAATCTCAGCAACTTTAG	
*SmMYC2b*sclF	AAAGATCTTATGGGGGTTGTTGGTTGG	Subcellecularlocalization (*SmMYC2b*)
*SmMYC2b*sclR	AAACTAGTCCCGAGAGATAACTGATG	
*SmMYC2a*peF	AAGGATCCATGATTGATTACCGCACGCC	Prokaryoticexpression (*SmMYC2a*)
*SmMYC2a*peR	AAGCTTCTATCTAATCTCAGCAACTTTAG	
*SmMYC2b*peF	AAGATATCATGGGGGTTGTTGGTTGG	Prokaryoticexpression (*SmMYC2b*)
*SmMYC2b*peR	AAGTCGACTTACCCGAGAGATAACTGATG	
*SmMYC2a*pADF	AACATATGATGATTGATTACCGCACGCC	Yeast-twohybrid (*SmMYC2a*)
*SmMYC2a*pADR	AAGAATTCCTATCTAATCTCAGCAACTTTAG	
*SmMYC2b*pADF	GGGAATTCCATATGGAATTCCCGATGGGGGTTGTTGGTTGG	Yeast-twohybrid (*SmMYC2b*)
*SmMYC2b*pADR	TCCCCCCGGGGGGATTACCCGAGAGATAACTGATG	
*SmJAZ1*pBDF	AAGAATTCATGGAGAGAGATTTCATGGG	Yeast-twohybrid (*SmJAZ1*)
*SmJAZ1*pBDR	AAGGATCCTCAGTCATCCTTGCTGACGG	
*SmJAZ2*pBDF	AACATATGATGTCTAGTTCAAGAATG	Yeast-twohybrid (*SmJAZ2*)
*SmJAZ2*pBDR	AAGAATTCTCATAATTTGAGCTCAAG	
*SmMYC2a*RNAiF	AATCTAGACCATGGGGGCTCCACGGAGCCCATCTTC	RNAi(*SmMYC2a*)
*SmMYC2a*RNAiR	AAGGATCCGGTACCAGACCTCTTCTTACTCTCCT	
*SmMYC2b*RNAiF	AATCTAGACCATGGCCTCAGGTCGAAACAAGC	RNAi(*SmMYC2b*)
*SmMYC2b*RNAiR	AAGGATCCGGTACCCCTTGAGGTCGCTCCAGTAAAATC	

Supplementary Table S3. Primers of *Sm*MYC2s and pathway genes used for qRT-PCR analysis.

Primer name	Sequence (5'→3')	Note
SmMYC2a RTF	CTAGGAAACGGGGAAGGAAG	qRT-PCR(*Sm*MYC2a)
SmMYC2a RTR	TGGACTTGAGCTCGTTGATG	
SmMYC2b RTF	GTTGCCAACGGGAATAGAG	qRT-PCR(*Sm*MYC2b)
SmMYC2b RTR	GCTTCCTCGGCCTCTTATC	
SmCMK RTF	CCTTGGTGGTGGCAGCAGTAA	qRT-PCR(*SmCMK*)
SmCMK RTR	GGTGGAGGAATATCTTCGACG	
SmDXR RTF	TGGCCAGAGAGAATCTACTGC	qRT-PCR(*SmDXR*)
SmDXR RTR	GGCTGCACTAAGAACTCCGGT	
SmHMGR RTF	ACGTGCGTGTTGCTACGAGAC	qRT-PCR (*SmHMGR*)
SmHMGR RTR	CGTCGCCAGTGCTGCAACTAA	
SmMCS RTF	CCGACGGTGATGTATTGCTG	qRT-PCR (*SmMCS*)
SmMCS RTR	GCATAGATTCGCCCGCATAG	
SmMK RTF	AGAGCATTGGTAGCTGGTGT	qRT-PCR (*SmMK*)
SmMK RTR	TCTCCATCAGCTCCCCTAGT	
SmPMK RTF	AGCACCTTGGAGAGCTCATT	qRT-PCR (*SmMK*)
SmPMK RTR	CTTCCGGCTTGCTCTTTTGT	
SmGPPS RTF	TTGTTACACCGCTGCGAAAA	qRT-PCR (*SmGPPS*)
SmGPPS RTR	ATGTTTGCCTTGCGATTGGT	
SmGGPPS RTF	TGAAATCACGGAAGCGCATT	qRT-PCR (*SmGGPPS*)
SmGGPPS RTR	TCGTGGATCATCGGTGGATT	
SmCPS RTF	ACTACCGTTCATCAAGGCCA	qRT-PCR (*SmCPS*)
SmCPS RTR	CCTCGAGTTGATTCTGCACG	
SmKSL RTF	AGAGGGCTCATGTCGAACAA	qRT-PCR (*SmKSL*)
SmKSL RTR	TTCTGCAGCCAATTGACACC	
SmPAL RTF	ACCTACCTCGTCGCCCTATGC	qRT-PCR (*SmPAL*)
SmPAL RTR	CCACGCGGATCAAGTCCTTCT	
SmC4H RTF	CCAGGAGTCCAAATAACAGAGCC	qRT-PCR (*SmC4H*)
SmC4H RTR	GAGCCACCAAGCGTTCACCAA	
Sm4CL RTF	ATTCGCATTCGCATTTCTCGG	qRT-PCR (*Sm4CL*)
Sm4CL RTR	GCGGCGTAGTGCTTCACCTTT	
SmTAT RTF	TTCAACGGCTACGCTCCAACT	qRT-PCR (*SmTAT*)
SmTAT RTF	AAACGGACAATGCTATCTCAAT	
SmHPPR RTF	GACTCCAGAAACAACCCACATT	qRT-PCR (*SmHPPR*)
SmHPPR RTR	CCCAGACGACCCTCCACAAGA	
SmHCT6 RTF	CCCTCCATTTCATCAGCACG	qRT-PCR (*SmRAS6*)

SmHCT6 RTR	GATTTGTCGGTGTTGGGGAG	
SmCYP98A14 RTF	CCATCATCGCCCTTCTTTGG	qRT-PCR (*SmCYP*)
SmCYP98A14 RTR	TGTCCACTTCCGTCATCACA	
Smactin F	ATGATAACTCGACGGATCGC	qRT-PCR (Housekeeping gene)
Smactin R	CTTGGATGTGGTAGCCGTTT	

Supplementary Table S4. Primers used in the identification of transgenic hairy roots.

Primer name	Sequence (5'→3')	Note
JDPDKF	ACAGTGGTCCCAAAGATGGA	Identification of vector pCambia1300-pHANNBIAL
JDPDKR	GGCGGTAAGGATCTGAGCTA	
HptF	CGATTTGTGTACGCCCGACAGTC	Identification of hairy roots
HptR	CGATGTAGGAGGGCGTGGATATG	
rolBF	GCTCTTGCAGTGCTAGATTT	Identification of hairy roots
rolBR	GAAGGTGCAAGCTACCTCTC	
rolCF	CTCCTGACATCAAACTCGTC	Identification of hairy roots
rolCR	TGCTTCGAGTTATGGGTACA	
